# Poly[tetra­aquadi-μ_4_-oxalato-lutetium(III)potassium]

**DOI:** 10.1107/S1600536811042061

**Published:** 2011-10-22

**Authors:** Feng-Ming Zhang, Tao-Zhu Sun, Guang-Feng Hou, Peng-Fei Yan, Guang-Ming Li

**Affiliations:** a Key Laboratory of Functional Inorganic Materials Chemistry (MOE), School of Chemistry and Materials Science, Heilongjiang University, Harbin 150080, People’s Republic of China

## Abstract

In the title compound, [KLu(C_2_O_4_)_2_(H_2_O)_4_]_*n*_, the Lu^III^ ion lies on a site of 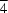 symmetry in a dodeca­hedron defined by eight O atoms from four oxalate ligands. The K atom lies on another site of the same symmetry and is coordinated by four oxalate O atoms and four O water atoms. The mid-point of the C—C bond of the oxalate group lies on an inversion center. In the packing structure, each oxalate ligand links two Lu(III) and two K atoms, forming a three-dimensional open framework with channels running along [001]. Inter­molecular O—H⋯O hydrogen bonds occur.

## Related literature

For background to oxalate anions as bridging ligands in high dimensional frameworks and for a similar structure, see: Camara *et al.* (2003 ▶); Zhang *et al.* (2009 ▶).
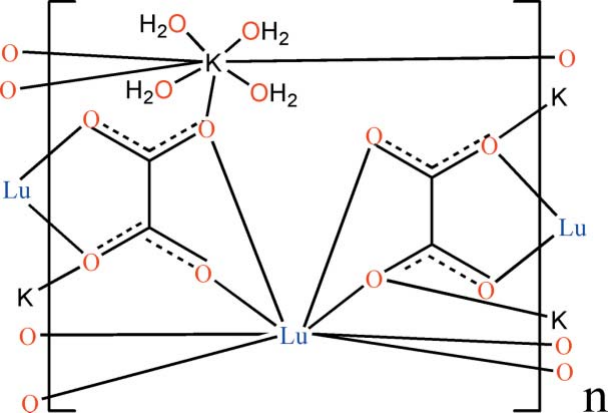


## Experimental

### 

#### Crystal data

[KLu(C_2_O_4_)_2_(H_2_O)_4_]
*M* *_r_* = 462.17
Tetragonal, 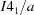

*a* = 11.3337 (16) Å
*c* = 8.9121 (18) Å
*V* = 1144.8 (3) Å^3^
*Z* = 4
Mo *K*α radiation
μ = 9.05 mm^−1^
*T* = 293 K
0.08 × 0.08 × 0.06 mm

#### Data collection

Rigaku R-AXIS RAPID diffractometer
Absorption correction: multi-scan (*ABSCOR*; Higashi, 1995 ▶) *T*_min_ = 0.546, *T*_max_ = 0.604
5421 measured reflections
655 independent reflections
594 reflections with *I* > 2σ(*I*)
*R*_int_ = 0.038

#### Refinement

*R*[*F* ^2^ > 2σ(*F* ^2^)] = 0.014
*wR*(*F* ^2^) = 0.035
*S* = 1.13
655 reflections
41 parameters
H-atom parameters constrained
Δρ_max_ = 0.64 e Å^−3^
Δρ_min_ = −0.40 e Å^−3^

### 

Data collection: *RAPID-AUTO* (Rigaku, 1998 ▶); cell refinement: *RAPID-AUTO*; data reduction: *CrystalClear* (Rigaku/MSC, 2002 ▶); program(s) used to solve structure: *SHELXS97* (Sheldrick, 2008 ▶); program(s) used to refine structure: *SHELXL97* (Sheldrick, 2008 ▶); molecular graphics: *SHELXTL* (Sheldrick, 2008 ▶); software used to prepare material for publication: *SHELXL97*.

## Supplementary Material

Crystal structure: contains datablock(s) I, global. DOI: 10.1107/S1600536811042061/ng5233sup1.cif

Structure factors: contains datablock(s) I. DOI: 10.1107/S1600536811042061/ng5233Isup2.hkl

Additional supplementary materials:  crystallographic information; 3D view; checkCIF report

## Figures and Tables

**Table 1:** Hydrogen-bond geometry (Å, °)

*D*—H⋯*A*	*D*—H	H⋯*A*	*D*⋯*A*	*D*—H⋯*A*
O3—H2⋯O1^i^	0.85	2.06	2.836 (4)	151
O3—H1⋯O3^ii^	0.85	2.06	2.891 (3)	166

Symmetry codes: (i) 
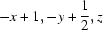
; (ii) 
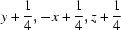
.

**Fig. 1.**
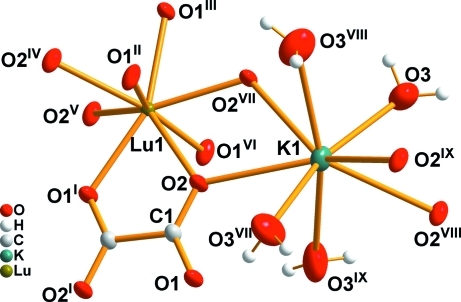
The molecular structure of the title compound, showing displacement ellipsoids at the 50% probability level for non-H atoms, Symmetry codes: (I) 1 - x, 1 - y, -z; (II) 1.25 - y, -1/4 + x, -1/4 + z; (III) x, -1/2 + y, -z; (IV) 0.25+Y, 0.75-X, -0.25-Z; (V) 0.75 - y, -1/4 + x, -0.25 - z; (VI) -1/4 + y, 0.75 - x, -1/4 + z; (VII) 1 - x, 0.5 - y, z; (VIII) 0.75 - y, -1/4 + x, 0.75 - z; (IX) 1/4 + y, 0.75 - x, 0.75 - z.

**Fig. 2.**
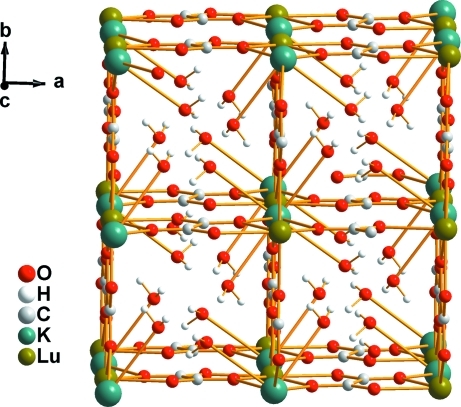
A partial packing view, showing the three-dimensional open framework along [001].

## supplementary crystallographic information

### Comment

Lanthanide complexes with spectroscopic and magnetic properties are currently of considerable interest; the oxalate ligand can serve as bridging ligand in high dimensional frameworks (Camara *et al.*, 2003; Zhang *et al.*, 2009). In this paper, we present here the synthesis and crystal structure of the title compound.

The title compound was obtained as a byproduct by the decomposition of 1,3,5-triazine-2,4,6-tricarboxylate ligand. In the title compound, [LuK(C_2_O_4_)_2_(H_2_O)_4_]_n_, the eight-coordinated lutetium(III) ion lies on a 4-fold inverse axis in a distorted dodecahedron defined by eight oxygen atoms from four oxalate ligands, and while the eight-coordinated potassium is also locate on a 4-fold inverseaxis in a distorted dodecahedron defined by four oxygen atoms from oxalate ligands and four oxygen atoms from water molecules (Fig. 1, Table 1).

In the packing structure, each oxalate ligand links two Lu(III) and two K atoms to form a three-dimensional open framework with channels running along [001] (Fig. 2).

### Experimental

The title compound was obtained as a byproduct caused by the decomposition of 1,3,5-triazine-2,4,6-tricarboxylate ligand. Lu(NO_3_)_3._6H_2_O (14.07 mg, 0.03 mmol) and the potassium salt of 1,3,5-triazine-2,4,6-tricarboxylate (9.8 mg, 0.03 mmol) were dissolved in 15 ml water. After stirring at room temperature for 0.5 h, the solution was allowed to stand for about one week; colorless block crystals were obtained in 40% yield.

### Refinement

Water H atoms were initially located in a differece Fourier map, but they were treated as riding on their parent atoms with O—H = 0.85 Å, and with *U*_iso_(H) = 1.5*U*_eq_(O).

### Crystal data

**Table:**

[KLu(C_2_O_4_)_2_(H_2_O)_4_]	*D*_x_ = 2.682 Mg m^−^^3^
*M**_r_* = 462.17	Mo *K*α radiation, λ = 0.71073 Å
Tetragonal, *I*4_1_/*a*	Cell parameters from 4696 reflections
Hall symbol: -I 4ad	θ = 3.1–27.5°
*a* = 11.3337 (16) Å	µ = 9.05 mm^−^^1^
*c* = 8.9121 (18) Å	*T* = 293 K
*V* = 1144.8 (3) Å^3^	Block, colorless
*Z* = 4	0.08 × 0.08 × 0.06 mm
*F*(000) = 872	

### Data collection

**Table:**

Rigaku R-AXIS RAPID diffractometer	655 independent reflections
Radiation source: fine-focus sealed tube	594 reflections with *I* > 2σ(*I*)
graphite	*R*_int_ = 0.038
ω scan	θ_max_ = 27.5°, θ_min_ = 3.6°
Absorption correction: multi-scan (*ABSCOR*; Higashi, 1995)	*h* = −13→14
*T*_min_ = 0.546, *T*_max_ = 0.604	*k* = −14→14
5421 measured reflections	*l* = −11→11

### Refinement

**Table:**

Refinement on *F*^2^	Primary atom site location: structure-invariant direct methods
Least-squares matrix: full	Secondary atom site location: difference Fourier map
*R*[*F*^2^ > 2σ(*F*^2^)] = 0.014	Hydrogen site location: inferred from neighbouring sites
*wR*(*F*^2^) = 0.035	H-atom parameters constrained
*S* = 1.13	*w* = 1/[σ^2^(*F*_o_^2^) + (0.0077*P*)^2^ + 3.796*P*] where *P* = (*F*_o_^2^ + 2*F*_c_^2^)/3
655 reflections	(Δ/σ)_max_ = 0.001
41 parameters	Δρ_max_ = 0.64 e Å^−^^3^
0 restraints	Δρ_min_ = −0.40 e Å^−^^3^

### Special details

**Table:**

Geometry. All e.s.d.'s (except the e.s.d. in the dihedral angle between two l.s. planes) are estimated using the full covariance matrix. The cell e.s.d.'s are taken into account individually in the estimation of e.s.d.'s in distances, angles and torsion angles; correlations between e.s.d.'s in cell parameters are only used when they are defined by crystal symmetry. An approximate (isotropic) treatment of cell e.s.d.'s is used for estimating e.s.d.'s involving l.s. planes.
Refinement. Refinement of *F*^2^ against ALL reflections. The weighted *R*-factor *wR* and goodness of fit *S* are based on *F*^2^, conventional *R*-factors *R* are based on *F*, with *F* set to zero for negative *F*^2^. The threshold expression of *F*^2^ > σ(*F*^2^) is used only for calculating *R*-factors(gt) *etc*. and is not relevant to the choice of reflections for refinement. *R*-factors based on *F*^2^ are statistically about twice as large as those based on *F*, and *R*- factors based on ALL data will be even larger.

### Fractional atomic coordinates and isotropic or equivalent isotropic displacement parameters (Å^2^)

**Table:**

	*x*	*y*	*z*	*U*_iso_*/*U*_eq_	
O3	0.3564 (3)	0.0435 (3)	0.4179 (4)	0.0588 (9)	
H2	0.3981	−0.0060	0.3698	0.088*	
H1	0.3356	0.0106	0.4995	0.088*	
O2	0.5074 (2)	0.36714 (18)	0.0938 (2)	0.0216 (5)	
O1	0.5037 (2)	0.55246 (18)	0.1842 (2)	0.0229 (5)	
C1	0.5028 (3)	0.4761 (3)	0.0803 (3)	0.0167 (6)	
K1	0.5000	0.2500	0.3750	0.0300 (3)	
Lu1	0.5000	0.2500	−0.1250	0.01106 (8)	

### Atomic displacement parameters (Å^2^)

**Table:**

	*U*^11^	*U*^22^	*U*^33^	*U*^12^	*U*^13^	*U*^23^
O3	0.081 (2)	0.0483 (19)	0.0472 (19)	0.0081 (17)	0.0202 (18)	−0.0078 (15)
O2	0.0371 (13)	0.0112 (9)	0.0164 (11)	−0.0010 (8)	−0.0012 (9)	0.0000 (8)
O1	0.0411 (13)	0.0127 (10)	0.0149 (10)	0.0009 (9)	−0.0012 (10)	−0.0011 (8)
C1	0.0198 (14)	0.0159 (14)	0.0143 (14)	−0.0008 (11)	−0.0012 (11)	0.0010 (11)
K1	0.0351 (5)	0.0351 (5)	0.0199 (7)	0.000	0.000	0.000
Lu1	0.01045 (9)	0.01045 (9)	0.01229 (13)	0.000	0.000	0.000

### Geometric parameters (Å, °)

**Table:**

O3—K1	2.876 (3)	K1—O2^iv^	2.837 (2)
O3—H2	0.8499	K1—O3^ii^	2.876 (3)
O3—H1	0.8500	K1—O3^iii^	2.876 (3)
O2—C1	1.242 (4)	K1—O3^iv^	2.876 (3)
O2—Lu1	2.361 (2)	Lu1—O1^v^	2.300 (2)
O2—K1	2.837 (2)	Lu1—O1^vi^	2.300 (2)
O1—C1	1.267 (4)	Lu1—O1^vii^	2.300 (2)
O1—Lu1^i^	2.300 (2)	Lu1—O1^i^	2.300 (2)
C1—C1^i^	1.531 (6)	Lu1—O2^viii^	2.361 (2)
K1—O2^ii^	2.837 (2)	Lu1—O2^ii^	2.361 (2)
K1—O2^iii^	2.837 (2)	Lu1—O2^ix^	2.361 (2)
			
K1—O3—H2	98.9	O2^iv^—K1—O3^iv^	120.99 (8)
K1—O3—H1	128.9	O2—K1—O3^iv^	97.09 (9)
H2—O3—H1	107.2	O3—K1—O3^iv^	91.015 (18)
C1—O2—Lu1	118.51 (19)	O3^ii^—K1—O3^iv^	91.015 (18)
C1—O2—K1	123.38 (18)	O3^iii^—K1—O3^iv^	164.71 (13)
Lu1—O2—K1	117.74 (8)	O1^v^—Lu1—O1^vi^	93.01 (2)
C1—O1—Lu1^i^	119.80 (19)	O1^v^—Lu1—O1^vii^	93.01 (2)
O2—C1—O1	127.4 (3)	O1^vi^—Lu1—O1^vii^	153.50 (11)
O2—C1—C1^i^	116.4 (3)	O1^v^—Lu1—O1^i^	153.50 (11)
O1—C1—C1^i^	116.2 (3)	O1^vi^—Lu1—O1^i^	93.01 (2)
O2^ii^—K1—O2^iii^	141.27 (5)	O1^vii^—Lu1—O1^i^	93.01 (2)
O2^ii^—K1—O2^iv^	141.27 (6)	O1^v^—Lu1—O2^viii^	81.67 (8)
O2^iii^—K1—O2^iv^	55.93 (8)	O1^vi^—Lu1—O2^viii^	69.06 (7)
O2^ii^—K1—O2	55.93 (8)	O1^vii^—Lu1—O2^viii^	137.39 (7)
O2^iii^—K1—O2	141.27 (6)	O1^i^—Lu1—O2^viii^	76.48 (8)
O2^iv^—K1—O2	141.27 (5)	O1^v^—Lu1—O2	137.39 (7)
O2^ii^—K1—O3	73.75 (8)	O1^vi^—Lu1—O2	81.67 (8)
O2^iii^—K1—O3	97.09 (9)	O1^vii^—Lu1—O2	76.48 (8)
O2^iv^—K1—O3	68.99 (8)	O1^i^—Lu1—O2	69.06 (7)
O2—K1—O3	120.99 (8)	O2^viii^—Lu1—O2	133.04 (6)
O2^ii^—K1—O3^ii^	120.99 (8)	O1^v^—Lu1—O2^ii^	69.06 (7)
O2^iii^—K1—O3^ii^	68.99 (8)	O1^vi^—Lu1—O2^ii^	76.48 (8)
O2^iv^—K1—O3^ii^	97.09 (9)	O1^vii^—Lu1—O2^ii^	81.67 (8)
O2—K1—O3^ii^	73.75 (8)	O1^i^—Lu1—O2^ii^	137.39 (7)
O3—K1—O3^ii^	164.71 (13)	O2^viii^—Lu1—O2^ii^	133.04 (6)
O2^ii^—K1—O3^iii^	97.09 (9)	O2—Lu1—O2^ii^	68.60 (10)
O2^iii^—K1—O3^iii^	120.99 (8)	O1^v^—Lu1—O2^ix^	76.48 (8)
O2^iv^—K1—O3^iii^	73.75 (8)	O1^vi^—Lu1—O2^ix^	137.39 (7)
O2—K1—O3^iii^	68.99 (8)	O1^vii^—Lu1—O2^ix^	69.06 (7)
O3—K1—O3^iii^	91.015 (18)	O1^i^—Lu1—O2^ix^	81.67 (8)
O3^ii^—K1—O3^iii^	91.015 (18)	O2^viii^—Lu1—O2^ix^	68.60 (10)
O2^ii^—K1—O3^iv^	68.99 (8)	O2—Lu1—O2^ix^	133.04 (6)
O2^iii^—K1—O3^iv^	73.75 (8)	O2^ii^—Lu1—O2^ix^	133.04 (6)

Symmetry codes: (i) −*x*+1, −*y*+1, −*z*; (ii) −*x*+1, −*y*+1/2, *z*; (iii) *y*+1/4, −*x*+3/4, −*z*+3/4; (iv) −*y*+3/4, *x*−1/4, −*z*+3/4; (v) *x*, *y*−1/2, −*z*; (vi) *y*−1/4, −*x*+3/4, *z*−1/4; (vii) −*y*+5/4, *x*−1/4, *z*−1/4; (viii) −*y*+3/4, *x*−1/4, −*z*−1/4; (ix) *y*+1/4, −*x*+3/4, −*z*−1/4.

### Hydrogen-bond geometry (Å, °)

**Table:**

*D*—H···*A*	*D*—H	H···*A*	*D*···*A*	*D*—H···*A*
O3—H2···O1^ii^	0.85	2.06	2.836 (4)	151.
O3—H1···O3^x^	0.85	2.06	2.891 (3)	166.

Symmetry codes: (ii) −*x*+1, −*y*+1/2, *z*; (x) *y*+1/4, −*x*+1/4, *z*+1/4.
